# Arbuscular Mycorrhizal Fungi Enhanced Drought Resistance of *Populus cathayana* by Regulating the 14-3-3 Family Protein Genes

**DOI:** 10.1128/spectrum.02456-21

**Published:** 2022-05-25

**Authors:** Yanyan Han, Xiao Lou, Wenrui Zhang, Tingying Xu, Ming Tang

**Affiliations:** a College of Forestry, Northwest A&F University, Yangling, People’s Republic of China; b Boone Pickens School of Geology, Oklahoma State University, Stillwater, Oklahoma, USA; c State Key Laboratory for Conservation and Utilization of Subtropical Agro-Bioresources, Guangdong Laboratory for Lingnan Modern Agriculture, College of Forestry and Landscape Architecture, South China Agricultural University, Guangzhou, People’s Republic of China; Beijing Forestry University

**Keywords:** antioxidant, arbuscular mycorrhizal fungi, drought stress, 14-3-3, osmotic regulation, *Populus cathayana*

## Abstract

Plants can improve their resistance to a variety of stresses by forming mutualistic relationships with arbuscular mycorrhizal fungi (AMF). The 14-3-3 protein is a major regulator of the plant stress response. However, the regulation mechanism of 14-3-3 family protein genes (14-3-3s) of mycorrhizal plants coping with stress during AMF symbiosis remains unclear. Here, we analyzed the physiological changes and 14-3-3 expression profiles of *Populus cathayana* inoculated with AMF under different water conditions. The results showed that good colonization and symbiotic relationships with plants were formed under all water conditions (63.00% to 83.67%). Photosynthesis, peroxidase (POD) activity, and Mg and Ca content were significantly affected by drought and AMF. In addition, thirteen 14-3-3 protein genes (*PcGRF1*-*PcGRF13*) were identified by quantitative real-time PCR (qRT-PCR), of which the expression levels of *PcGRF10* and *PcGRF11* induced by AMF were significantly positively correlated with superoxide dismutase (SOD), POD, and sugar content, indicating that the 14-3-3s of mycorrhizal symbiotic plants may respond to drought through antioxidant and osmotic regulation. This is the first study on 14-3-3s in the symbiosis system of forest arbor plants and AMF, and it may help to further study the effects of 14-3-3s during AMF symbiosis on stresses and provide new ideas for improving mycorrhizal seedling cultivation under stress.

**IMPORTANCE** The 14-3-3 protein may regulate many biochemical and physiological processes under abiotic stress. Studies have shown that the 14-3-3 protein gene of AMF is not only upregulated under drought stress, but also enhances the regulation of AMF on plant drought tolerance by regulating plant signal pathways and drought response genes; however, knowledge about the biological relevance of these interactions remains limited and controversial. The precise functions of *Populus cathayana* 14-3-3s under drought stress remain poorly resolved and the mechanisms of action of these genes in mycorrhizae-induced drought stress are still unknown. Thus, studying the drought-resistance mechanism of the AMF symbiotic plant 14-3-3 gene is of special significance to improving the drought tolerance of the plant. Further systematic study is needed to probe the mechanism by which AMF regulates different 14-3-3 genes and their subsequent physiological effects on drought.

## INTRODUCTION

As a limiting condition for plant survival, drought stress caused by excessive water deficiency can induce a wide range of physiological and transcriptional regulatory responses in plants and inhibit their growth and development. Nevertheless, plants have evolved a series of mechanisms to resist the adverse effects of drought stress at the cell, tissue, and whole-plant levels (1), which may show differences in morphology, growth rate, tissue penetration potential, antioxidant response, and hormone regulation (2). For example, a root system that loses water produces the ABA signal, which is transported to the overground parts through the xylem and then regulates the stomata closing (3); the concentration of intracellular osmotic regulatory substances increases to maintain the fullness and volume of the cell and resist drought (4).

In addition to internal systems that protect plants from biotic and abiotic stresses, plants can also establish beneficial relationships with some microorganisms in the rhizosphere (5), mitigating the damage caused by drought stress. Among these, arbuscular mycorrhizal fungi (AMF) symbiosis is an effective way to help terrestrial plants survive in adverse conditions. The external hyphae network of mycorrhizae can significantly promote the absorption of water and nutrients (6), improve photosynthesis (7), and regulate metabolic processes (8) to enhance the drought tolerance of the host plants. In addition, AMF can also increase plant resistance to drought stress by activating the expression of plant genes (9). Studies have shown that the 14-3-3 protein gene of AMF is not only upregulated under drought stress but also enhances the regulation of AMF on plant drought tolerance by regulating plant signal pathways and drought-response genes (10).

The 14-3-3 protein is a highly conserved protein with diverse functions which exists in almost all eukaryotes. It is widely distributed in organelles such as the cytoplasm, cell membrane, mitochondria, and chloroplasts. As a “bridge” between proteins, it regulates many biochemical and physiological processes under abiotic stress (11). The 14-3-3 isoform in plants was named GF14 (G-box Factor 14-3-3 homologs) or GRF (G-box Regulatory Factor or General Regulatory Factor) since it was identified as a part of a protein/G-box complex (12). The 14-3-3 family protein genes have been identified in more than 20 plants, including *Arabidopsis* (13 genes), rice (8), tomato (12), and mango (16) (13–16). In poplar, twelve 14-3-3 genes have been identified by genomic bioinformatic measurements based on the Populus trichocarpa genome V1.1 in previous studies (17). Tian et al. (18) further revealed two additional 14-3-3 genes in poplars and extended the total number to fourteen. In cotton, the overexpression of the *Arabidopsis* 14-3-3 gene *AtGF14k* resulted in improved water-stress tolerance (19). In contrast, overexpression of the *Glycine soja* 14-3-3 gene *GsGF14o* in Arabidopsis thaliana resulted in decreased drought tolerance during seed germination and seedling growth (20). Besides the contradictory results from previous studies, the precise functions of *Populus* 14-3-3s under drought stress remain poorly resolved, and the mechanism of these genes induced by AMF under drought stress is still unknown. Previous studies have confirmed that the responses of 14-3-3 protein genes to drought stress in mycorrhizal symbiotic systems are mainly involved in the regulation of ABA signaling pathways (21) and the cross-linking of d-*myo*-inositol-3-phosphate synthase (22). Thus, a further systematic study is needed to probe the mechanism by which AMF regulate different 14-3-3 genes and its subsequent physiological effects on drought resistance.

Populus cathayana Rehd. (*P. cathayana*) is a kind of dioecious plant with rapid growth which plays an important role in afforestation and ecological protection in northern China (23–27), and male plants are more resistant than female plants to environmental stresses such as drought and salt. Previous studies have determined that the 14-3-3 genes from AMF itself or from the plants induce drought resistance (28, 29). We hypothesized that either (i) the 14-3-3s play an important role and are differentially expressed in *P. cathayana* inoculated with AMF under drought stress, or (ii) the 14-3-3s induced by AMF are related to the physiological indicators of *P. cathayana* under drought stress. Based on these, we characterized plant responses to drought stress during the mycorrhization process using a multidisciplinary approach focusing on photosynthesis, antioxidant systems, osmotic adjustment, and expression of 14-3-3 protein family stress-response genes in plants and AMF. The aim of this study was to provide new ideas to improve drought resistance in mycorrhizal seedlings.

## RESULTS

### Mycorrhizal colonization.

**FIG 1:**
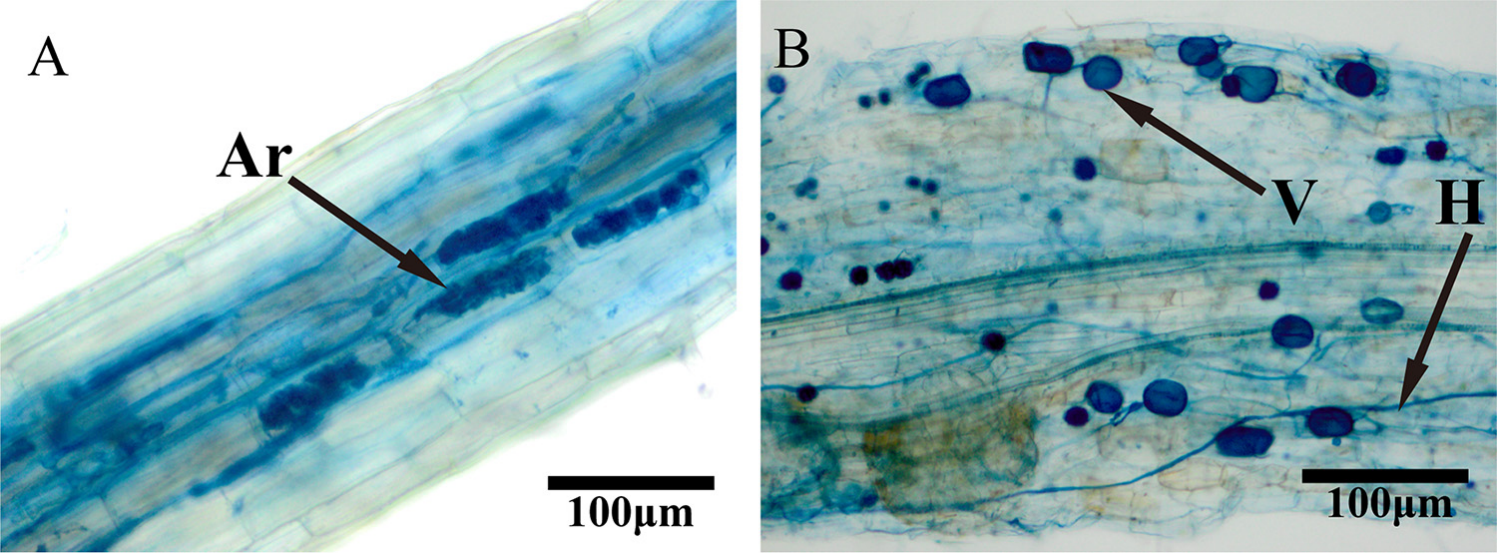
Typical structure of arbuscular mycorrhizal fungi (AMF) in the root system of *Populus cathayana*. Ar, arbuscule; V, vesicle; H, hypha. Scale bar = 100μm.

The root system of *P. cathayana* inoculated with AMF formed a typical mycorrhizal structure under different water conditions (Fig. 1), with total colonization rates of 72.67% for “well-watered” (WW), 83.67% for “mild drought” (MD), and 63.00% for “extreme drought” (ED). Under ED conditions, no arbuscular structure was observed, and hyphal structure was significantly reduced by 57.08% compared with that of the WW group. Arbuscule formation was increased by 100.12% and hypha colonization rate was only increased by 6.83% under the MD condition compared to WW (Table 1).

**TABLE 1:** Mycorrhizal colonization rates of *P. cathayana* under different drought conditions^*a*^

Treatment	AMF structural colonization (%)			Total
	Arbuscule	Vesicle	Hypha	
AM + WW	8.33 ± 7.37b	57.67 ± 10.50a	68.33 ± 7.64a	72.67 ± 5.51ab
AM + MD	16.67 ± 4.04a	62.00 ± 6.08a	73.00 ± 6.08a	83.67 ± 5.67a
AM + ED	0	63.67 ± 7.09a	29.33 ± 2.89b	63.00 ± 7.00b

a AMF, arbuscular mycorrhizal fungi; WW, well-watered condition; MD, mild drought condition; ED, extreme drought condition; AM, mycorrhizal treatment with *R. intraradices*. Matching lowercase letters following the means indicate nonsignificant differences between corresponding treatments as determined by Duncan’s multiple range test (*P* < 0.05). Results are shown as mean ± standard deviation (*n* = 3).

### Plant height, RWC, and root vitality.

**FIG 2:**
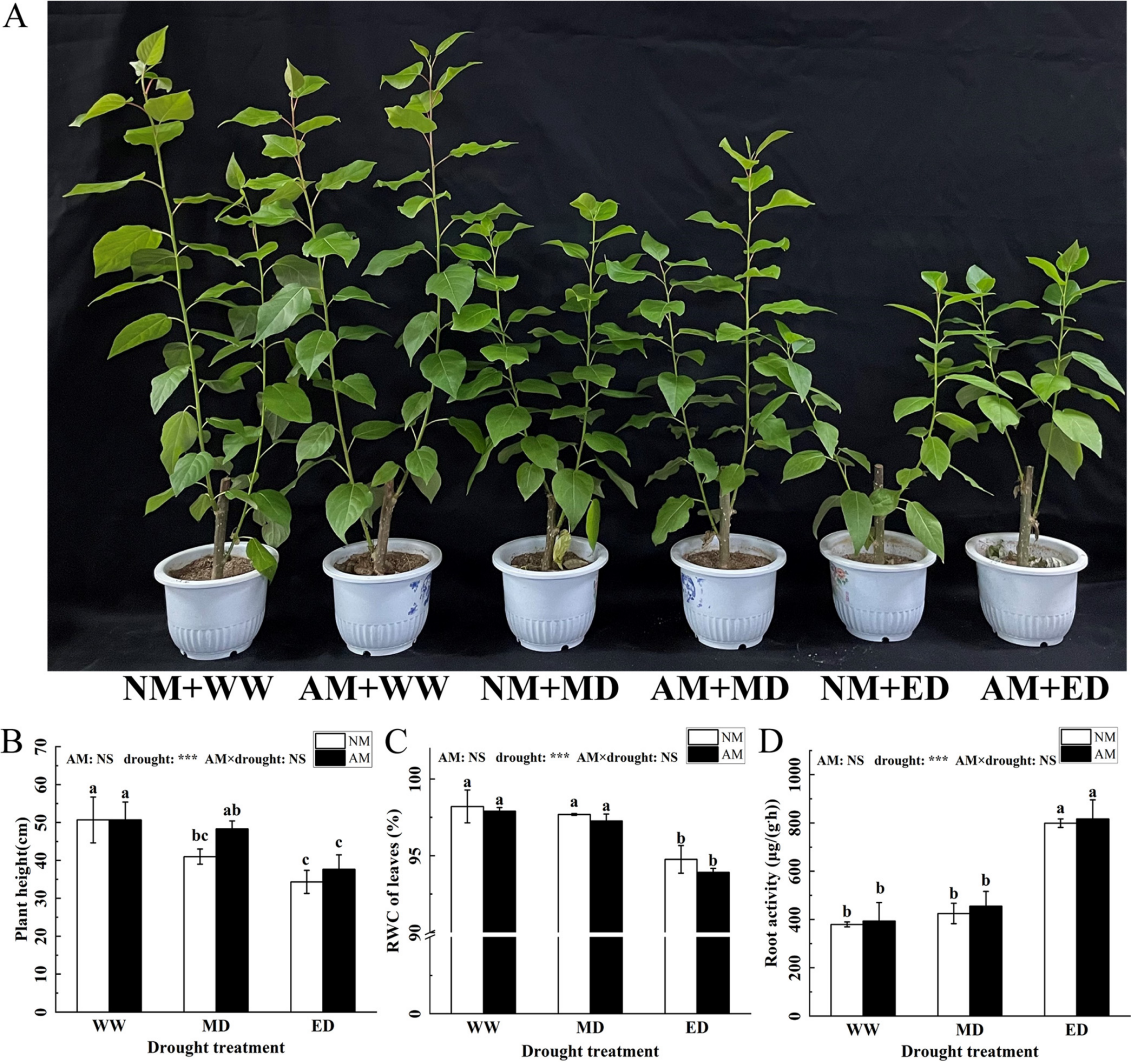
Growth picture (A), plant height (B), relative water content (RWC) (C), and root activity (D) of *P. cathayana* under different drought conditions. (mean ± standard deviation [SD], *n* = 3). WW, well-watered; MD, mild drought; ED, extreme drought condition; NM, non-mycorrhizal treatment; AM, mycorrhizal treatment with Rhizophagus intraradices. Columns marked by the same lowercase letters are not significantly different according to Duncan’s multiple range test (*P* < 0.05). Significance of two-way analysis of variance (ANOVA): *, *P* < 0.05; **, *P* < 0.01; ***, *P* < 0.001; NS, not significant.

The height of plants in the mycorrhizal treatment (AM) group was significantly higher than that of plants in the non-mycorrhizal (NM) group under MD stress, but which were decreased by 19.08% (NM) and 4.62% (AM) compared to that under the WW condition (Fig. 2B). Plant height and leaf relative water content in *P. cathayana* seedlings significantly decreased and root activity significantly increased under ED conditions (Fig. 2).

### Plant biomass.

**FIG 3:**
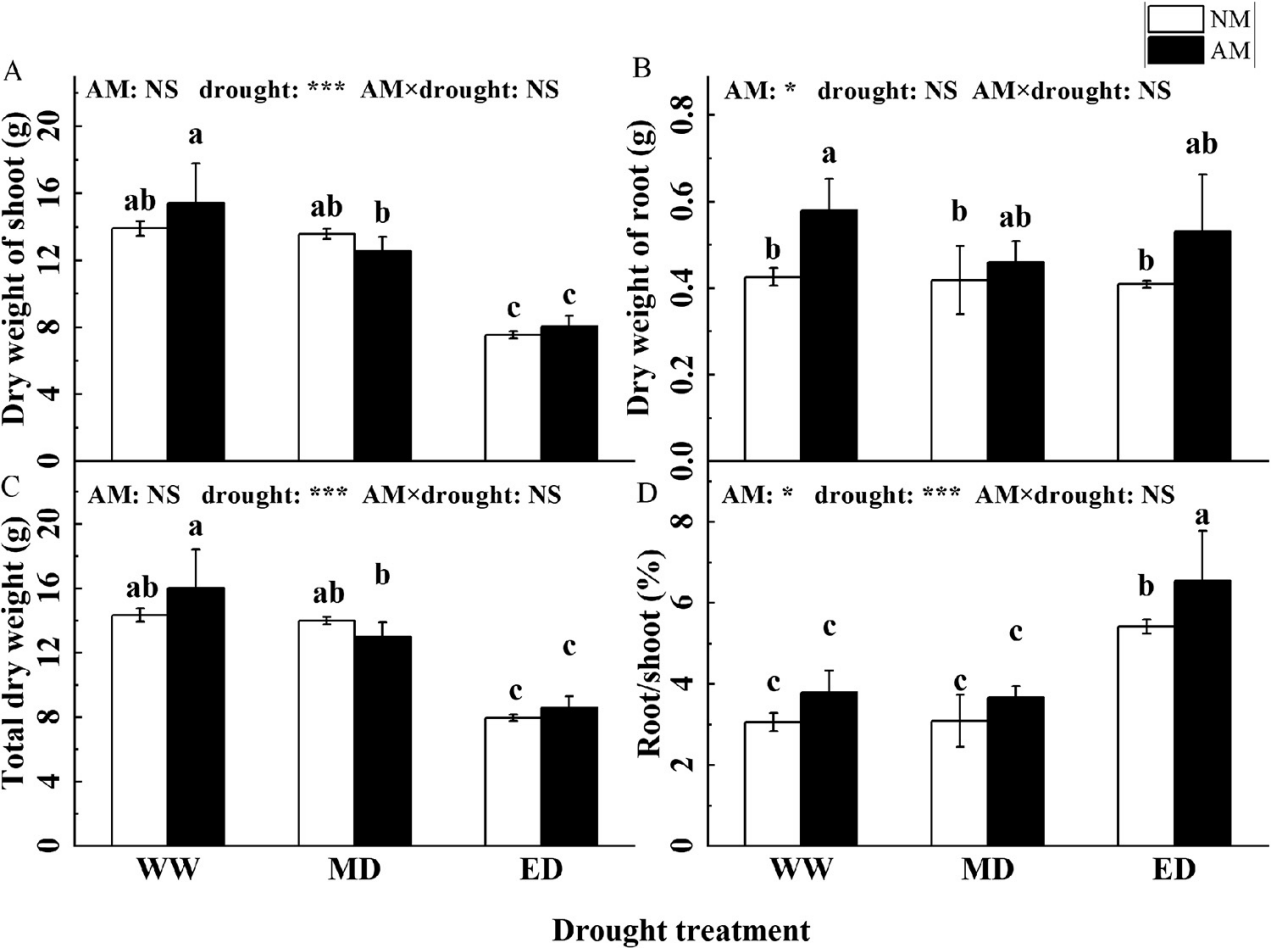
Effect of AMF inoculation on the biomass of *P. cathayana* under drought stress (mean ± SD, *n* = 3).

Mycorrhizal inoculation significantly increased root biomass (WW: 36.07%, MD: 9.79%; ED: 29.70%) and root-to-shoot ratio (ED: 20.66%). The shoot biomass of *P. cathayana* was significantly reduced and the root-to-shoot biomass ratio was significantly increased under ED conditions (Fig. 3).

### Photosynthesis.

**FIG 4:**
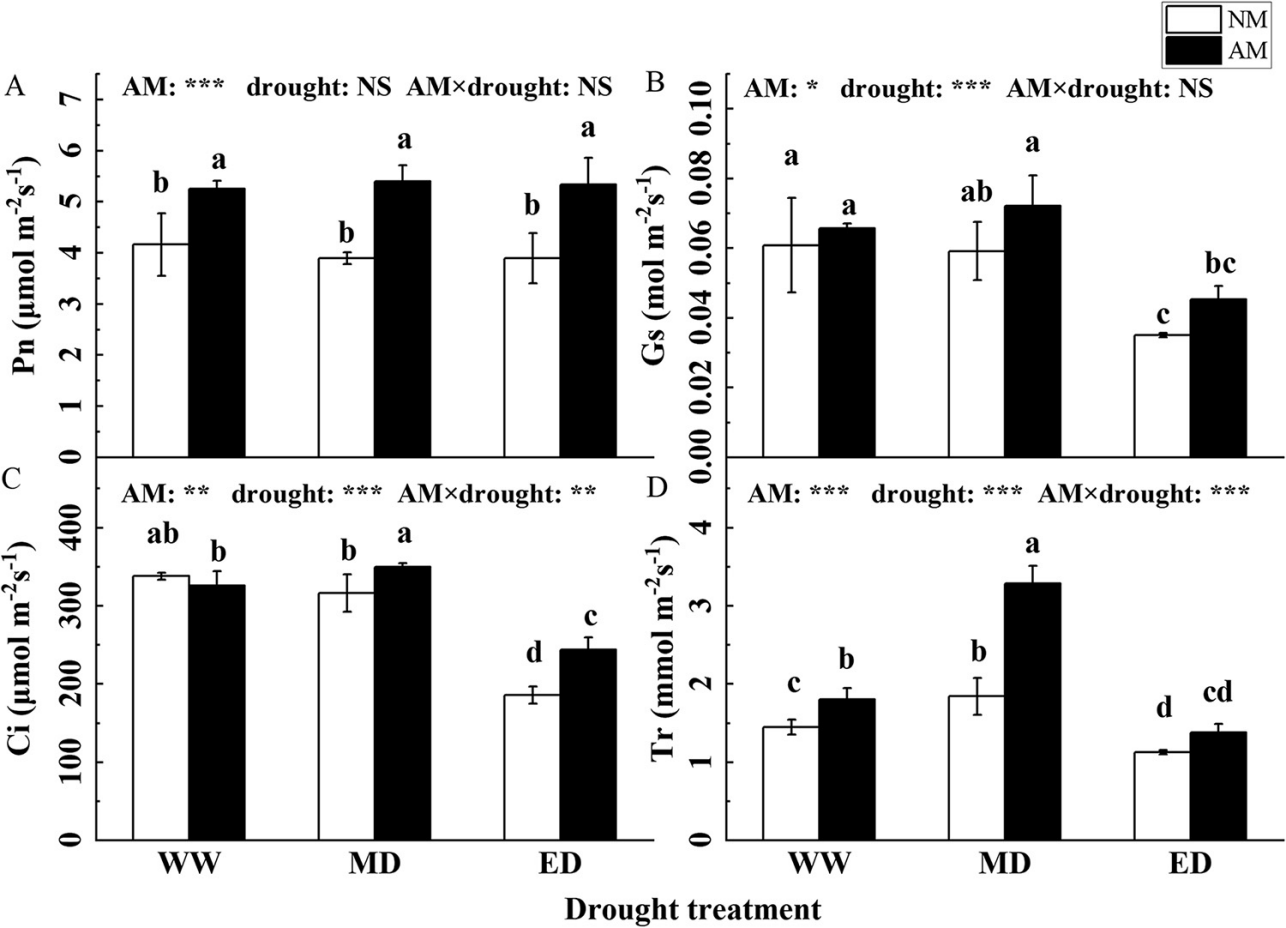
Effect of AMF inoculation on the photosynthesis of *P. cathayana* under drought stress (mean ± SD, *n* = 3).

The levels of stomatal conductance (Gs), intercellular CO_2_ concentration (Ci), and transpiration rate (Tr) were significantly decreased under ED conditions. However, the leaf photosynthetic rates (Pn) in the NM and AM groups were not decreased under drought conditions, and the Pn of the AM group was significantly increased by 26.34% for WW, 38.87% for MD, and 37.04% for ED compared to that of the NM group. Photosynthesis was significantly improved by AMF inoculation, with increased or significantly increased Pn, Gs, Ci, and Tr (Fig. 4).

### Antioxidant activity.

**FIG 5:**
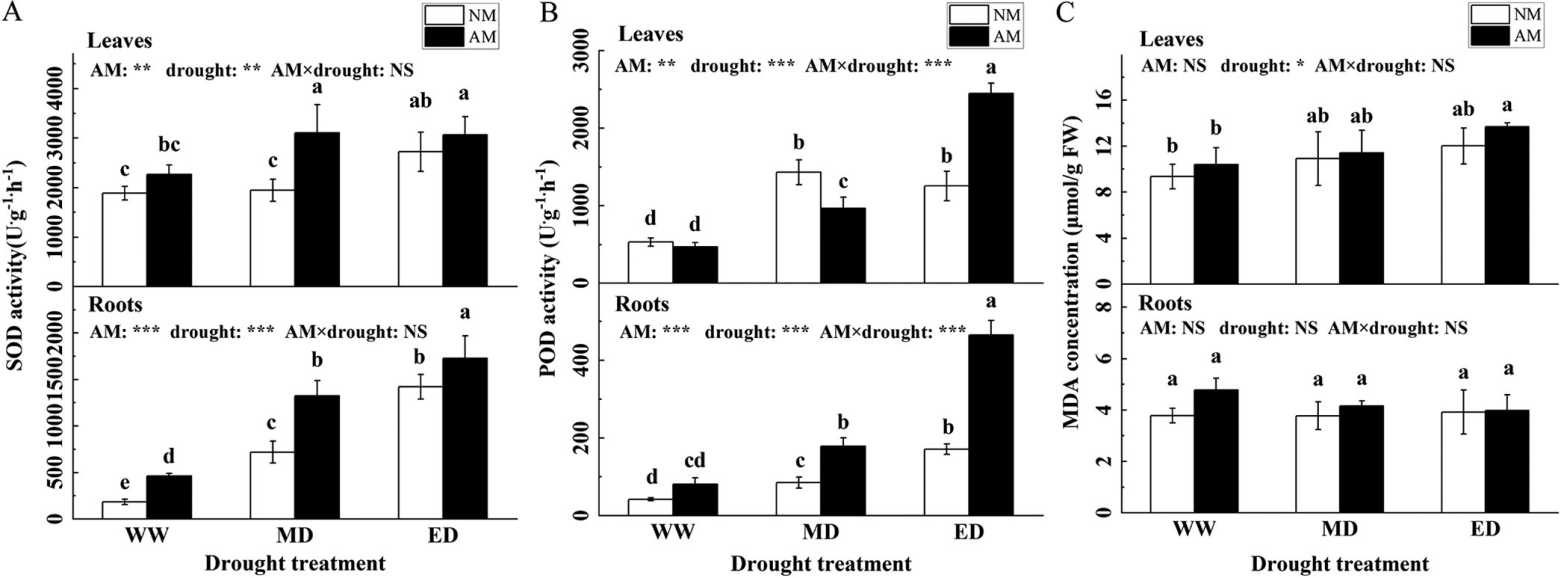
Effect of AMF inoculation on the antioxidant activity of *P. cathayana* under drought stress (mean ± SD, *n* = 3).

Mycorrhizal inoculation resulted in 94.81% and 171.68% increases in peroxidase (POD) activity in the leaves and roots compared to that of the non-inoculation group under the ED condition. However, superoxide dismutase (SOD) activity in leaves and roots inoculated with AMF increased the most under the MD and WW conditions, increasing by 59.83% and 149.04%, respectively, compared to that in the NM treatment group (Fig. 5A and B). The malondialdehyde (MDA) concentration of the leaf was mainly influenced by drought stress, but that of both the leaf and the root was generally not responsive to mycorrhizal inoculation (Fig. 5C).

### Soluble substance.

The soluble sugar content increased with the increase in drought levels, especially for the leaves which, in the AM group, had significantly higher content than those in the NM group under the three water conditions. Soluble protein, starch, and cellulose content varied inconsistently in the leaves and roots. AMF inoculation had a promoting effect on soluble protein content in roots under ED conditions, cellulose content in the root, and starch content in leaves under WW conditions (Fig. S1 in the supplemental material).

### Nutrient element content.

K content decreased and P content increased with increased drought (Fig. S2A and B). P content in the root was significantly improved by AMF inoculation, increasing by 84.9% (WW), 52.7% (MD), and 35.7% (ED) compared to that of the non-inoculation group. Mycorrhizal inoculation significantly increased the Ca (13.97% under ED conditions) and Mg content (16.84% under MD and 22.02%, ED conditions) of leaves compared to that in the non-inoculation group (Fig. S2C and D).

### Principal-component analysis and orthogonal partial least-squares discrimination analysis of physiological indicators.

**FIG 6:**
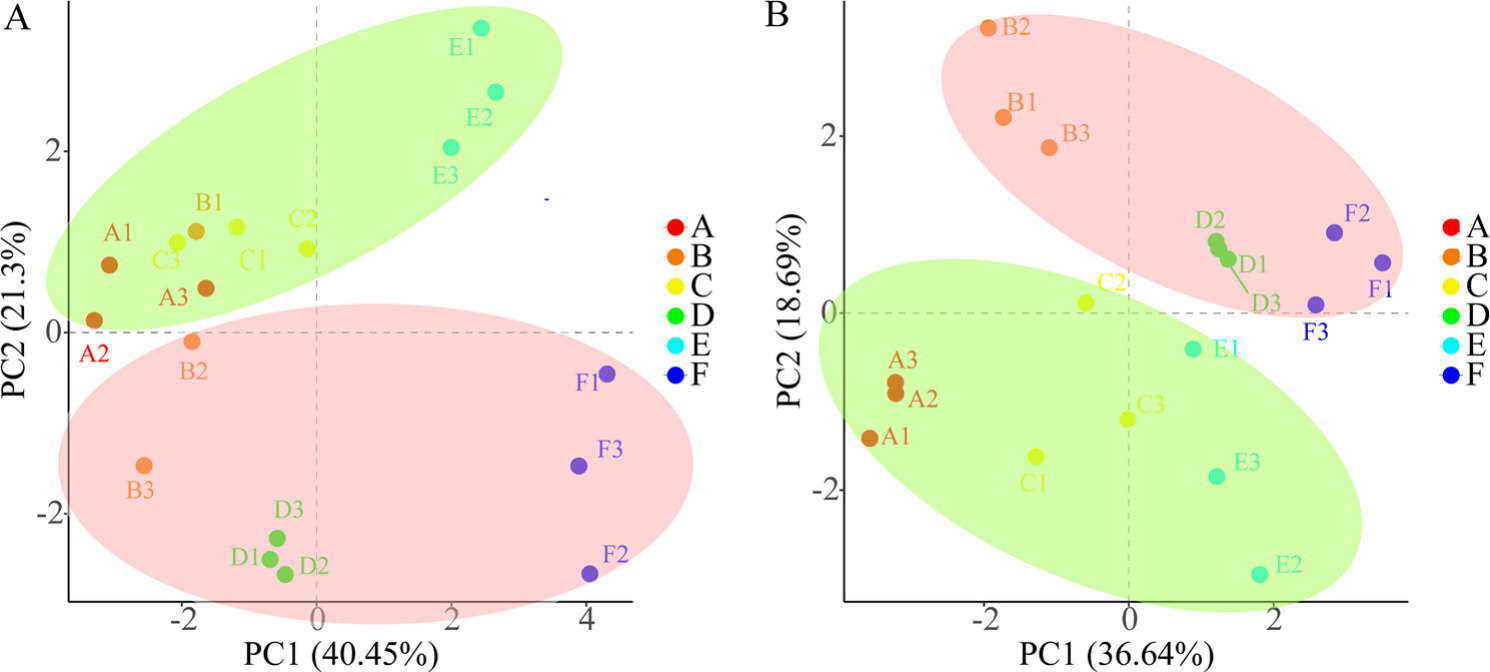
Principal-component analysis (PCA) results (panel A: leaf; panel B: root) of physiological indices of *P. cathayana* under different drought and inoculation conditions. PC1, the first principal component; PC2, the second principal component. Red A, NM + WW; orange B, AM + WW; yellow C, NM + MD; green D, AM + MD; light blue E, NM + ED; dark blue F, AM + ED.

In Fig. 6A, PC1 explained 40.45% of the total variable and PC2 explained 21.30%. In Fig. 6B, PC1 and PC2 explained 36.64% and 18.69% of the total variable, respectively. The drought groups (AB, CD, and EF) and inoculation groups (AM: BDF and NM: ACE) could be distinguished relatively clearly in terms of first principal component (PC1) and second principal component (PC2), respectively (Fig. 6A and B). In general, the six groups had a high degree of differentiation in both leaves and roots. Therefore, the responses of these variables to drought were greater than the effects of mycorrhizal inoculation in both the leaf and the root. In Fig. 6A, the distance between EF and the other four treatments was notable, indicating that extreme drought had a great influence on the physiological indicators of leaves.

**FIG 7:**
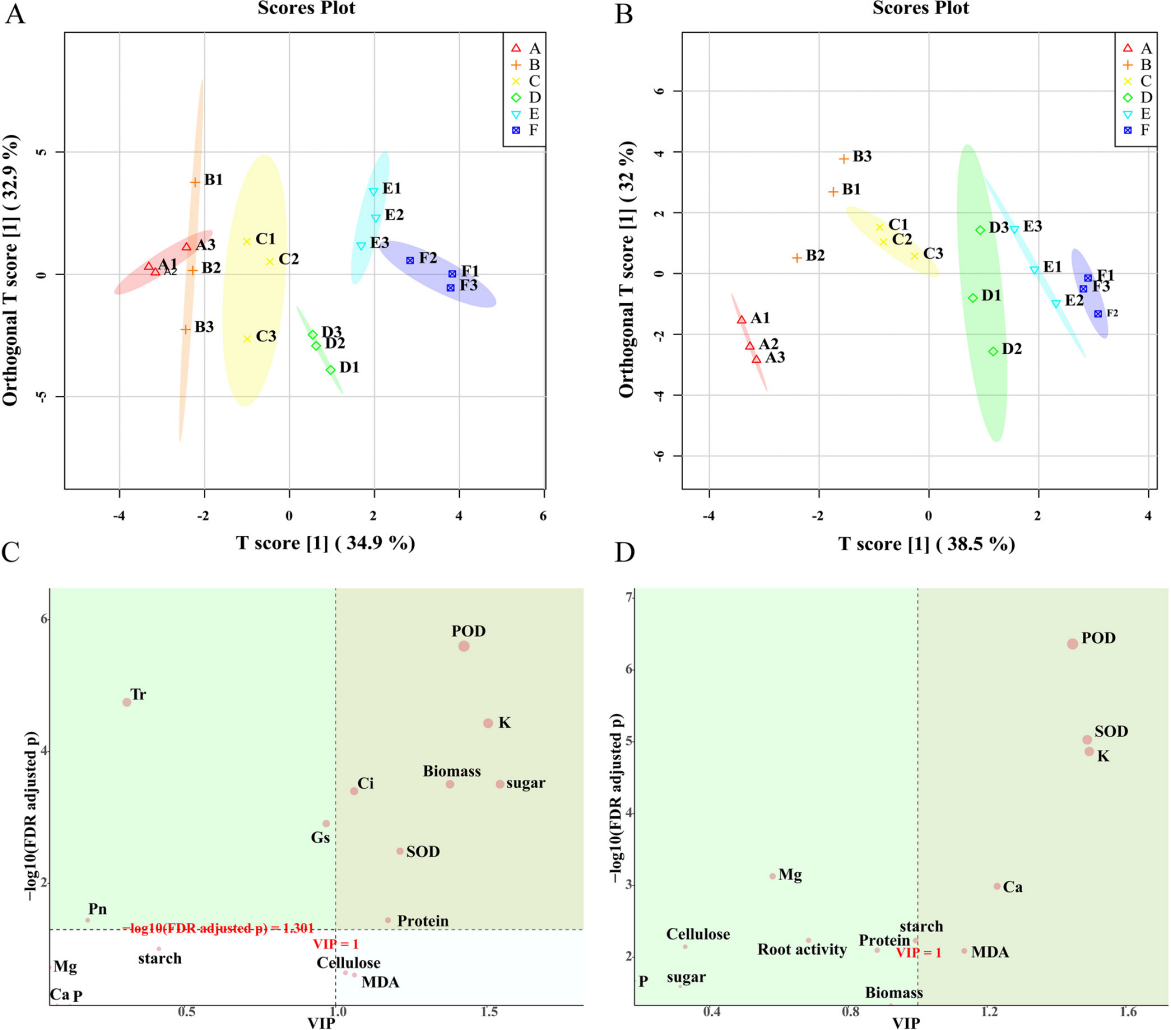
Orthogonal partial least-squares discrimination analysis (OPLS-DA) results (panels A and C: leaf; panels B and D: root) of physiological indices of *P. cathayana* under different drought and inoculation conditions. Red A (triangles), NM + WW; orange B (+), AM + WW; yellow C (×), NM + MD; green D (diamonds), AM + MD; light blue E (inverted triangles), NM + ED; dark blue F (checked boxes), AM + ED.

According to the orthogonal partial least-squares discrimination analysis (OPLS-DA) (Fig. 7), the data for the 6 groups could be easily distinguished (Fig. 7A and B). The main substances which produced differences in leaves were POD, K, biomass, sugar, Ci, SOD, and protein (Fig. 7C, VIP > 1, FDR < 0.05). Only POD, K, Ci, and SOD were affected by both drought and mycorrhiza. Additionally, POD, SOD, K, Ca, and MDA were the primary different metabolites in roots between the AM group and NM groups (Fig. 7D, variable importance in projection [VIP] > 1, false discovery rate [FDR] < 0.05), and POD and SOD were also affected by drought.

### Relative expression of the *P. cathayana* 14-3-3 gene family.

**FIG 8:**
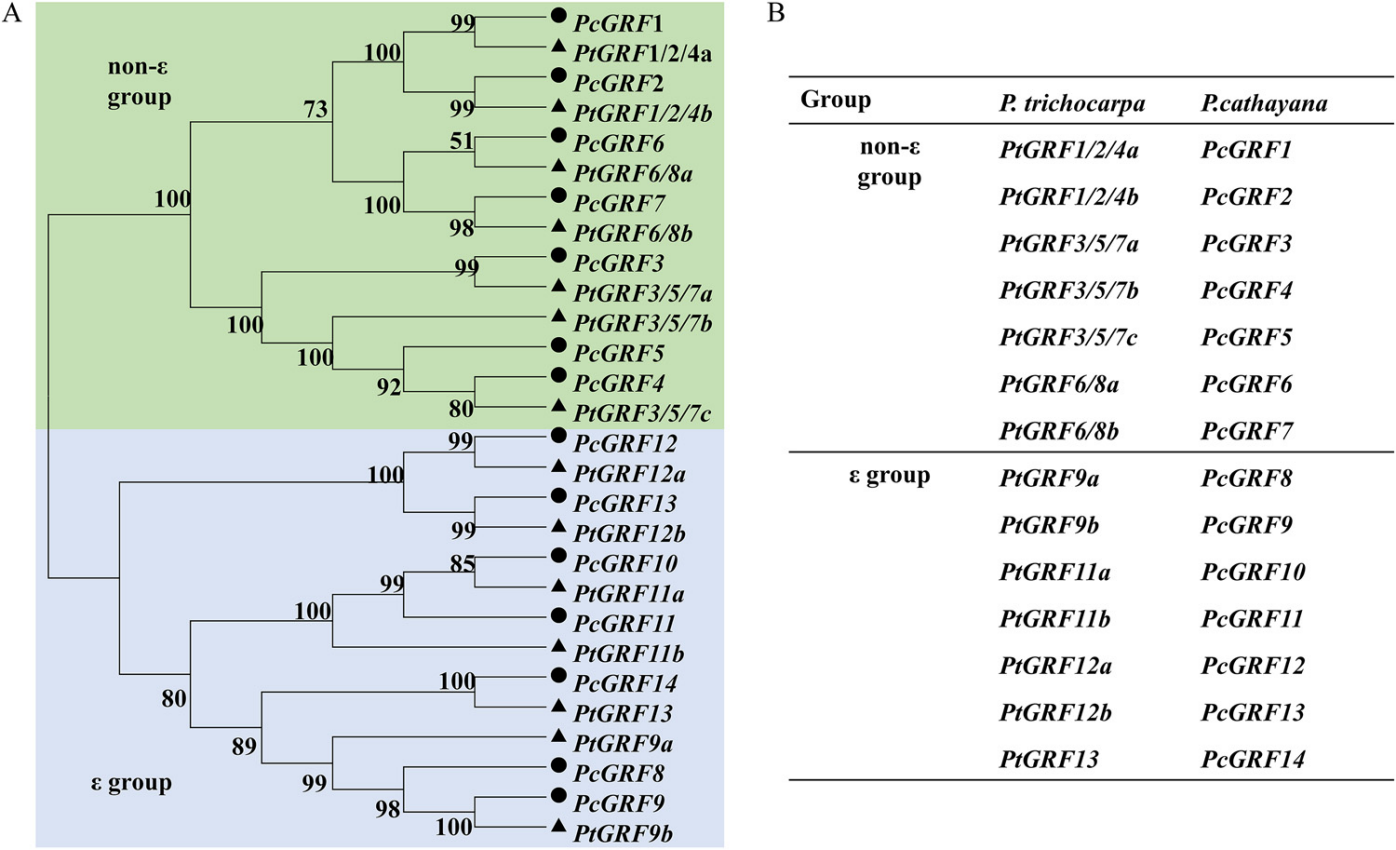
Phylogenetic analysis (A) and gene-name comparison (B) of 14-3-3 gene family in *P. cathayana* and *P. trichocarpa*. The two major groups are marked with different background colors.

**FIG 9:**
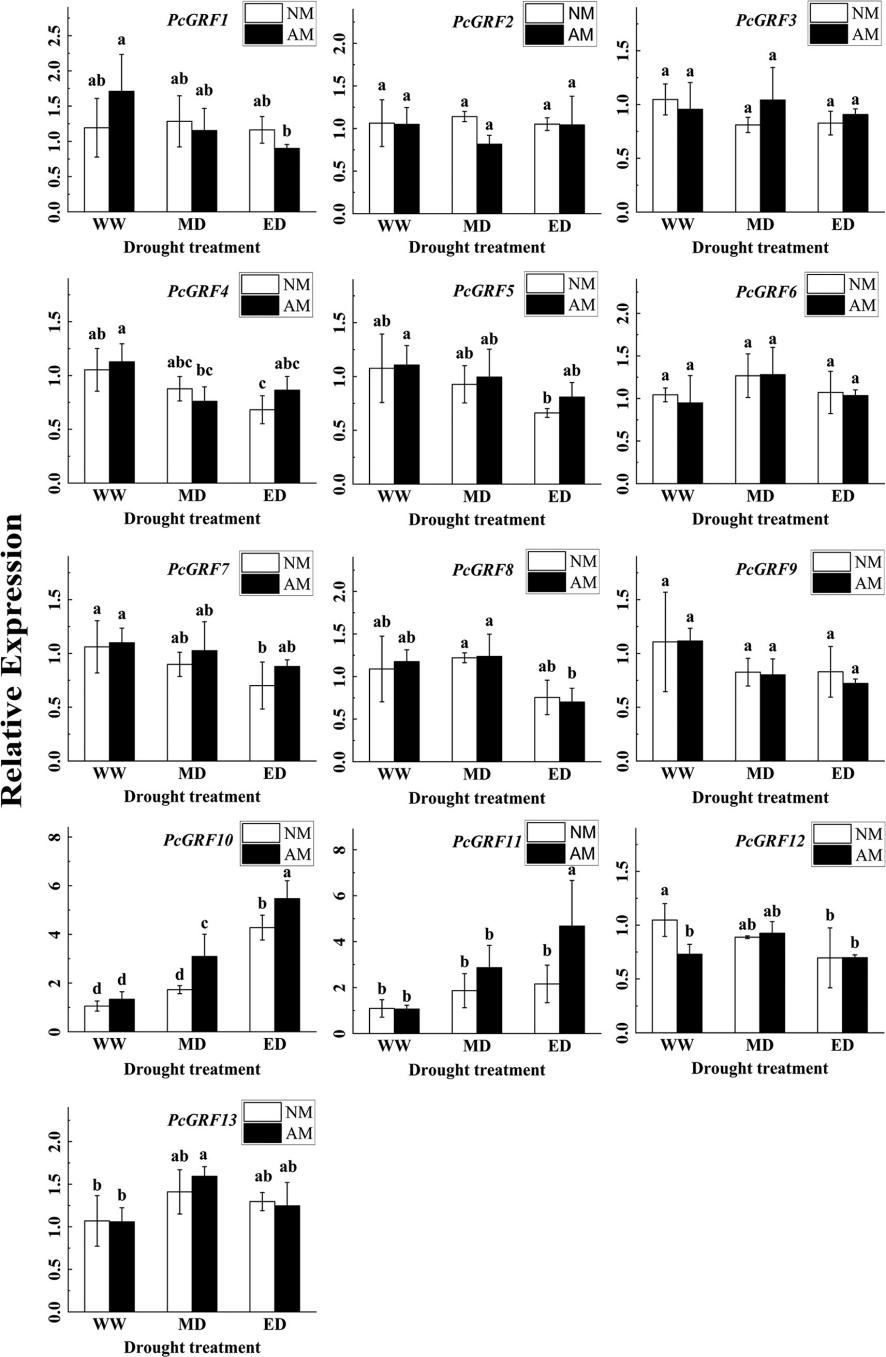
Relative expression of 14-3-3 protein genes in leaves of *P. cathayana* under different water conditions (mean ± SD, *n* = 3).

**FIG 10:**
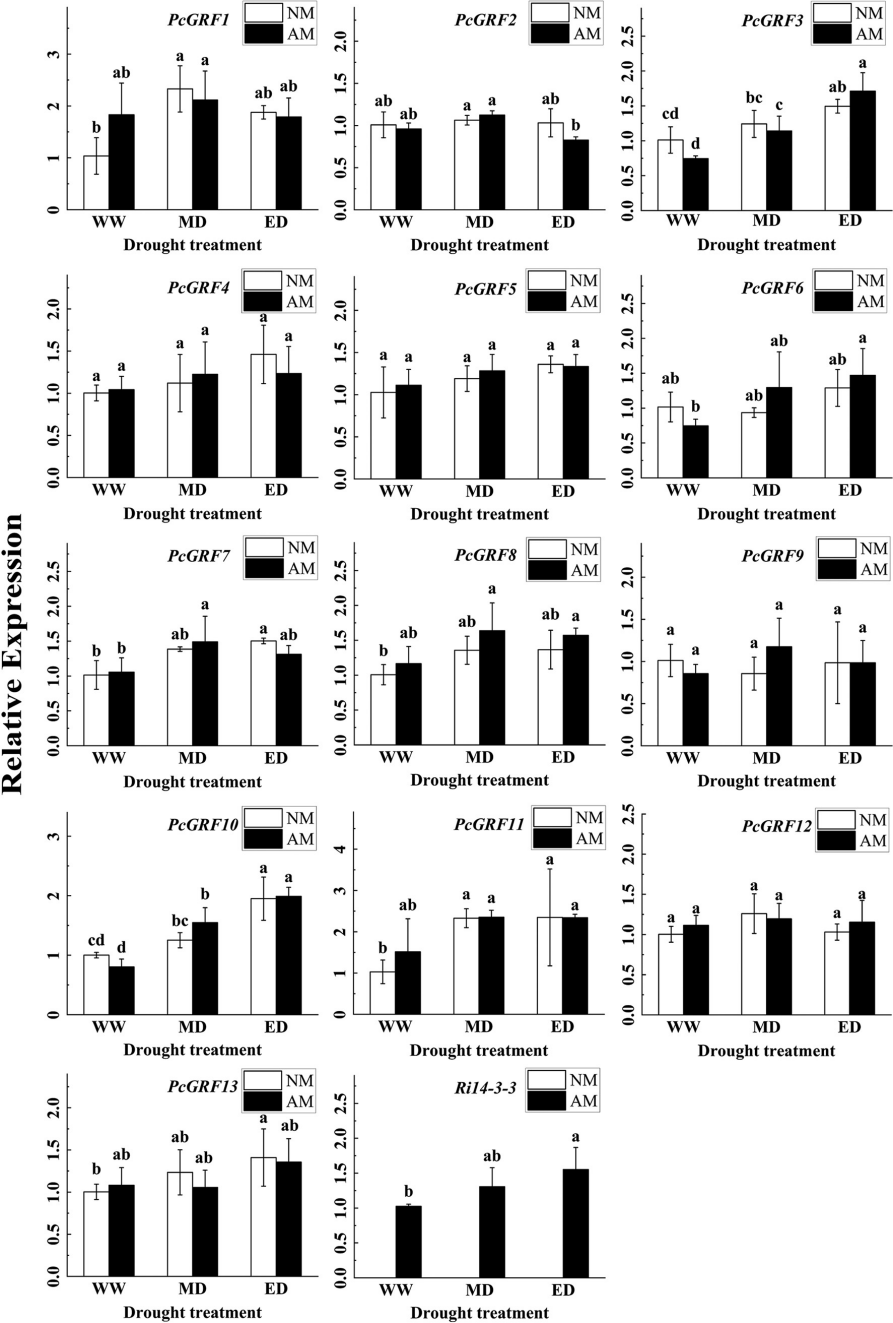
Relative expression of 14-3-3 protein genes in roots of *P. cathayana* and *R. intraradices* under different water conditions (mean ± SD, *n* = 3).

We totally cloned fourteen sequences of 14-3-3 genes in *P. cathayana* according to the *P. trichocarpa* 14-3-3 gene family (Fig. 8A and B), but only thirteen (*PcGRF1*-*PcGRF13*) of them were identified by quantitative real-time PCR (qRT-PCR). The 14-3-3 protein family genes sequence of *P. cathayana* (*PcGRF1-PcGRF14*) has been submitted to the GenBank database of NCBI, GenBank accession numbers are ON236646-ON236659. The expression levels of the thirteen 14-3-3 genes in leaves and roots were analyzed by RT-PCR, as shown in Fig. 9 and 10.

The expression of *PcGRF10* and *PcGRF11* in *P. cathayana* leaves was significantly upregulated in response to drought and AMF. The relative expression of *PcGRF10* in the NM group increased by 63.88% and 305.44% under the MD and ED conditions compared to that under WW conditions, and *PcGRF11* expression increased by 70.91% and 97.67%, respectively. Under MD and ED conditions, the relative expression of *PcGRF10* in the AM group increased by 78.75% and 27.81% compared to that in the NM group, and *PcGRF11* increased by 53.58% and 117.05%, respectively. However, the expression of *PcGRF10* and *PcGRF11* in roots was significantly upregulated only under the induction of drought. The relative expression of *PcGRF10* in the NM group increased by 25.07% and 94.88% under the MD and ED conditions compared to that under WW conditions, and *PcGRF11* increased by 126.38% and 128.18%. The expression of *Ri14-3-3* was significantly upregulated by drought and continued to be upregulated with the increase in drought.

### OPLS-DA analysis of gene expression.

**FIG 11:**
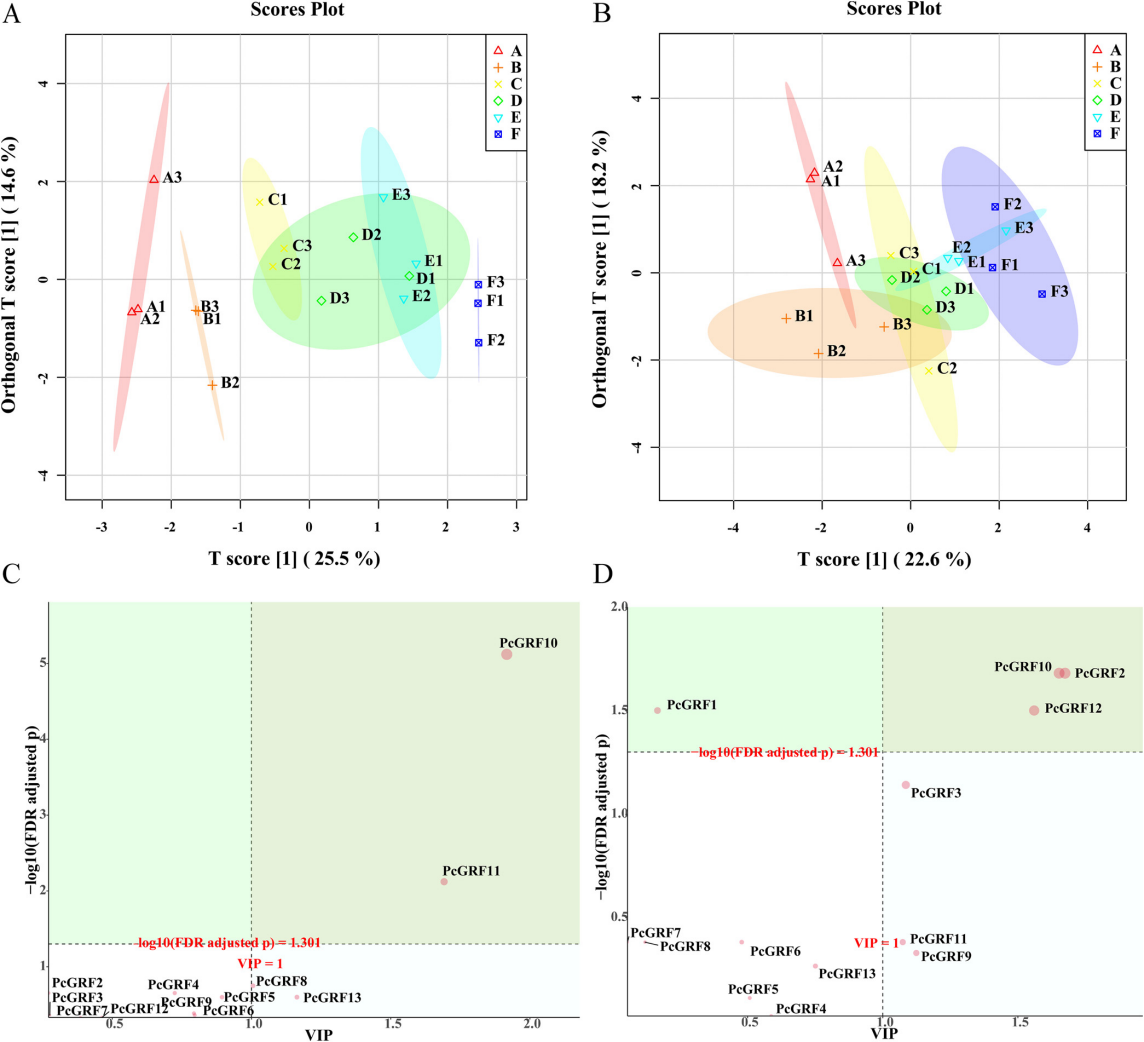
OPLS-DA results (panels A and C: leaf; panels B and D: root) of gene expression in *P. cathayana* under different drought and inoculation conditions. Red A (triangles), NM + WW; orange B (+), AM + WW; yellow C (×), NM + MD; green D (diamonds), AM + MD; light blue E (inverted triangles), NM + ED; dark blue F (checked boxes), AM + ED.

According to OPLS-DA (Fig. 11), the 6 data sets were not well distinguished (Fig. 11A and B). *PcGRF10* and *PcGRF11* (VIP > 1, FDR < 0.05) were the main differentially expressed genes in leaves for drought and mycorrhizal inoculation response (Fig. 11C). *PcGRF10*, *PcGRF2*, and *PcGRF12* (VIP > 1, FDR < 0.05) were the main differentially expressed genes in roots for drought response (Fig. 11D).

### Correlation analysis of 14-3-3s and physiological indicators.

The expression of *Ri14-3-3* was significantly upregulated by drought (Fig. 10). Correlation analysis was conducted between the plant 14-3-3 gene in the inoculation group and the expression level of *Ri14-3-3* in the mycorrhiza. We found that the expression levels of *PcGRF10* and *PcGRF11* in leaves and *PcGRF3*, *PcGRF4*, *PcGRF6*, *PcGRF8*, *PcGRF10*, and *PcGRF11* in roots were positively correlated with *Ri14-3-3* expression levels (Fig. S3). Correlation analysis of the plant 14-3-3 gene and physiological indicators showed that the common differential physiological indicators (POD, SOD, and K) had consistent correlations with *PcGRF10* and *PcGRF11* in both the leaf and root (Fig. S4).

**FIG 12:**
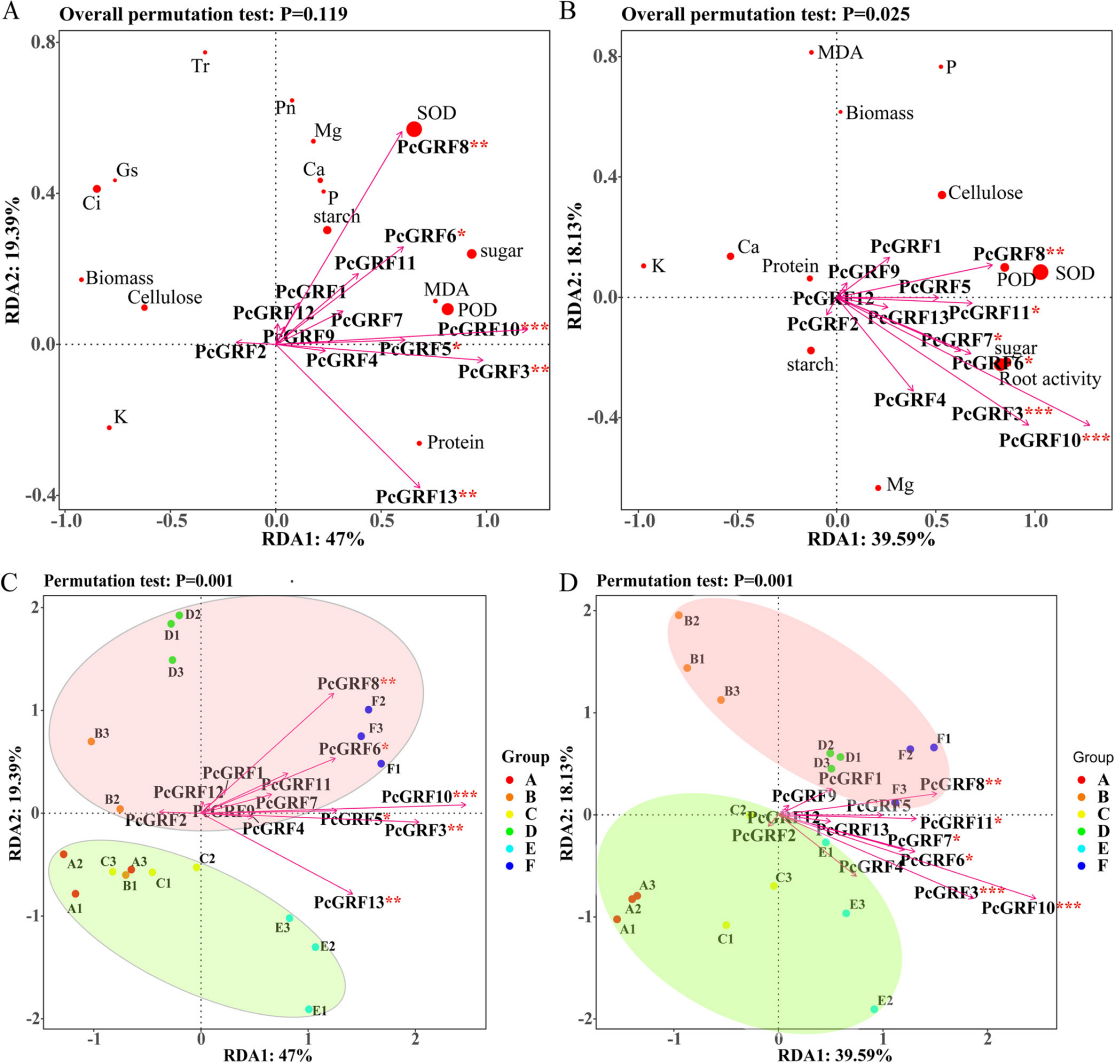
Redundancy analysis (RDA) results (panels A and C: leaf; panels B and D: root) of gene expression and physiological indicators of *P. cathayana* under different drought and inoculation conditions. Red A, NM + WW; orange B, AM + WW; yellow C, NM + MD; green D, AM + MD; light blue E, NM + ED; dark blue F, AM + ED. *, *P* < 0.05; **, *P* < 0.01; ***, *P* < 0.001.

The arrows of genes in the leaves and roots were almost at acute angles with the first sorting axis RDA1 and pointed to E and F and their intermediate region, indicating that the 14-3-3s were regulated in response to drought. *PcGRF3*, *PcGRF5*, *PcGRF6*, *PcGRF8*, *PcGRF10*, and *PcGRF13* (*P* < 0.05) were the main genes which affected physiological metabolism for drought response in leaves, and *PcGRF3*, *PcGRF6*, *PcGRF7*, *PcGRF8*, *PcGRF10*, and *PcGRF11* (*P* < 0.05) were the main ones that affected the drought metabolites in roots. The genes (*P* < 0.01) which had an acute angles with RDA1, including *PcGRF3*, *PcGRF8*, *PcGRF10*, and *PcGRF13* in leaves and *PcGRF3*, *PcGRF8*, and *PcGRF10* in roots, may be more likely to be expressed in mycorrhizal plants under drought conditions than under non-arid conditions (Fig. 12C and D). *PcGRF10* may be the main gene affecting the physiological indicators of drought resistance of *P. cathayana* because it had the longest projection length on the first ordination axis in both leaves and roots (Fig. 12).

## DISCUSSION

### AM symbiosis improved physiological activity in plants under drought conditions.

In this research, we used Rhizophagus intraradices, an AMF capable of establishing symbiotic relationships with many plants, to inoculate *P. cathayana* seedlings. Our results showed that the development of mycorrhizae is most favorable when soil water content is 50% ~ 60% of the maximum field water capacity, consistent with the findings of Lu et al. (30) and Liu et al. (31). Under ED conditions, *P. cathayana* seedlings had a low hypha colonization rate and high vesicle formation, but the formation of arbuscules was not observed. It may be that arbuscules appeared during the initial stage of AMF colonization in plant roots, then gradually died out and became granular, and extreme drought accelerated their extinction; or that AMF chose a self-protection mechanism under extreme water shortage conditions, since a similar mechanism has been proposed in which severe water stress can cause woody plants to stop growing completely and enter a dormant state (32).

AMF can absorb water and nutrients from soil areas which cannot be reached by roots by forming a mycelial bridge in the roots to maintain the normal physiological and metabolic functions of plants under drought stress (33). Photosynthesis, as the basis of plant metabolism, is the most important chemical reaction in plants. Studies have shown that AMF symbiosis can alleviate the negative effects of drought stress on net photosynthesis and transpiration rates (34, 35). We found that mycorrhizal inoculation increased or significantly increased the photosynthesis of *P. cathayana* under different water conditions. The negative effects of photosynthesis may be due to the destruction of photosynthetic organs by ROS (reactive oxygen species) generated by biological stress (36). The POD and SOD activities in *P. cathayana* increased with the increase of drought degree, indicating that plants can reduce the accumulation of intracellular ROS and its inhibition of photosynthesis by increasing the production of ROS-scavenging enzymes (37). *P. cathayana* inoculated with AMF may enhance its drought tolerance by accumulating soluble sugar and cellulose content (38). In general, *P. cathayana* coped with drought stress by enhancing antioxidant enzyme activity and regulating osmotic potential.

### Effects of AMF on 14-3-3 family protein gene expression.

Systematically understanding the process of mycorrhizal fungus formation and its promotion of plant growth is of great importance to the reciprocal symbiosis of plants and AMF. In our study, *R. intraradices* was used to inoculate *P. cathayana* seedlings, as it is one of the few AMF with transcriptome data and can establish symbiotic relationships with many plants (39). According to previous studies, the 14-3-3 gene of AMF plays an important regulatory role in the early stage of mycorrhizal symbiosis (40) and drought-stress resistance (28). Porcel et al. (28) revealed that 14-3-3 protein gene *Ri14-3-3* of *R. intraradices* may be involved in AMF symbiosis to protect host plants against drought stress through studying the expression pattern of *Ri14-3-3* under 25% polyethylene glycol (PEG)-6000 drought stress *in vitro* when natural symbiosis was formed with different host plants. In our study, we found that the *Ri14-3-3* gene could be amplified by RNA extracted from the roots of inoculated AMF but not by RNA from the roots of non-inoculated plants. *Ri14-3-3* expression increased with increased drought and was positively correlated with the expression level of the 14-3-3 gene (*PcGRF10* and *PcGRF11*) in *P. cathayana* in both roots and leaves, indicating that the *Ri14-3-3* gene may be involved in physiological regulation and expression of drought-response genes of *P. cathayana* inoculated with AMF under drought stress.

Previous studies have indicated that the 14-3-3 family protein genes are an important factor and have allotype specificity for plants to respond to drought stress (29, 41–43). Therefore, it is necessary to examine the 14-3-3 protein gene family members involved in drought-stress response regulation in different plants. Tian et al. (18) identified and analyzed 14-3-3s of *P. trichocarpa*, the model for woody transgenic plants, conducting 14-3-3s expression pattern experiments in six different vegetative tissues by qRT-PCR, and they could not detect *PtGRF13*. This is consistent with our results. Our study found that the 14-3-3 genes (*PcGRF10* and *PcGRF11*) of *P. cathayana* may be mycorrhiza-induced genes under drought stress, consistent with the findings of Tian et al. (18) regarding the 14-3-3 partial response genes *PtGRF11a* and *PtGRF11b* of *P. trichocarpa* under abiotic stresses such as low nitrogen restriction, methyl jasmonate (MeJ) treatment, mechanical damage, hypoxia, and high temperature. *P. cathayana* and *P. trichocarpa* both belong to the *Tacamahaca* section, so their 14-3-3 genes could have similar expression profiles, as indicated by this work and previous studies. In general, mycorrhizal symbiosis could modify the response of plants to drought by upregulating 14-3-3 genes, preventing the reduction of plant water use efficiency and enhance the plants’ ability to cope with drought stress.

### Regulation of 14-3-3 family genes on physiological responses and protein targets.

14-3-3 proteins are key regulatory molecules prevalent in many metabolic and physiological pathways regulated by phosphorylation. In our research, root activity was enhanced, and the root-shoot biomass ratio was significantly increased, especially under the inoculation condition, which may be due to 14-3-3 proteins regulating root development to cope with stress (41, 44). It was found that the total root length, surface area, and volume of transgenic *AtGRF9* tomato were significantly increased (45), and the improved root development promoted the uptake of nutrients by plants (46, 47). In the higher plants, 14-3-3 proteins could activate plasma membrane H^+^-ATPase and expel protons from roots, which play a key role in maintaining root expansion and nutrient dissolution under abiotic stress, and can help plants obtain P (48, 49). Furthermore, plant H^+^-ATPase activity is also regulated by mycorrhizal colonization (50). It is known that mycorrhizal inoculation improves plant growth by promoting phosphorus acquisition (51). Therefore, the absorption of P by mycorrhizal plants under drought stress may have a synergistic effect with the 14-3-3 protein. The 14-3-3 protein also plays a role in the calcium signaling pathway under drought stress. Calcium-dependent protein kinases can directly convert intracellular Ca^2+^ signals into reversible phosphorylation events of various substrates, then mediate the interaction with 14-3-3 proteins to enhance drought tolerance in plants (52). This process may occur in the roots of *P. cathayana* which produce the major differential nutrient element Ca. In addition, the differential regulation of the encoding 14-3-3 gene subtypes in *Arabidopsis* and tomato induced by P, K, or Fe deficiency suggests that they are involved in multiple nutrient-sensing pathways (53–55).

The carbon newly fixed in plants during photosynthesis is used for cellular respiration and metabolism, instantaneously stored in vacuoles as sucrose or in plastids as starch. 14-3-3 protein is thought to be involved in carbohydrate metabolism and transport (56, 57). Studies have shown that overexpression of 14-3-3 protein genes leads to increased soluble sugar content (45), which participates in osmotic regulation and ROS signaling regulation under abiotic stress. Water acquisition can also be affected by the regulation of sucrose transport in the phloem (41). Previous studies have found that 14-3-3 protein genes could enhance the response of antioxidant systems to drought stress and induce the expression of ROS elimination system genes, which to some extent explains the decrease in ROS toxicity under drought conditions (58–60). Yan et al. (19) showed that 14-3-3 interacts with ascorbate peroxidase 3, a key enzyme which removes H_2_O_2_ and prevents damage under oxidative stress or water-deficiency conditions. Our study also found that antioxidant enzymes (SOD and POD), as the main differential metabolic enzymes of drought and AMF inoculation, may also be related to the expression of 14-3-3 differential genes (*PcGRF10* and *PcGRF11*). In summary, the 14-3-3 genes and AMF have many of the same functions, so we believe that AMF has an encouraging influence on the function of the 14-3-3 genes in AMF-inoculated plants. In general, the 14-3-3 protein genes in mycorrhizal *P. cathayana* seedlings may respond to drought stress by enhancing the antioxidant system and osmotic regulation. A complex 14-3-3 protein regulatory network exists in plants. The exact mechanisms by which 14-3-3 proteins regulate metabolism through these intricate interactions, and how cells transduce and fine-tune target proteins, remains elusive (61). Our study lays the foundation for the mechanism of 14-3-3 gene function in the mycorrhizal symbiosis system.

### Conclusions.

In summary, the 14-3-3 genes in *P. cathayana* responding to drought stress under mycorrhizal induction were mainly *PcGRF10* and *PcGRF11*, which could resist drought stress mainly by improving the antioxidant enzyme system (SOD and POD) and osmotic regulation of substances (soluble sugar and K). Our study provides preliminary information on the effects of AMF colonization on the expression of 14-3-3 protein family genes in *P. cathayana* under drought stress. which may help to further study the effects of 14-3-3s during AMF symbiosis under disadvantageous conditions and provide new ideas for improving mycorrhizal seedling cultivation under stress.

## MATERIALS AND METHODS

### Plant materials, culture substrate treatment, and AMF inoculation.

The experimental plants were annual male cuttings (length: 19 cm, diameter: 1 ~ 1.3 cm) of *P. cathayana*, purchased from the Qinghai Datong Linbo Seedling Professional Cooperative. The cuttings were surface-disinfected with 0.05% potassium permanganate and then soaked in sterile distilled water for use.

The sand was passed through a 2-mm sieve, washed, and sterilized in an oven at 170°C for 3 h, and vermiculite was sterilized in a high-pressure steam sterilizer at 121°C for 2 h. Sand and vermiculite were mixed in equal volume (vol:vol = 1: 1) and put into plastic pots (upper diameter: 12.5 cm, bottom diameter: 8 cm, height: 10 cm) for use.

R. intraradices was obtained from the Beijing Academy of Agriculture and Forestry Sciences (Beijing, China) and propagated with corn (Zea mays L.) in the laboratory of the College of Forestry, Northwest A&F University (Yangling, China). A mixture of spores (12 per g of inoculum), hyphae, and root segments was obtained as the test inoculum.

### Experimental design and treatments.

The experiment included two factors: inoculation treatment (AM: with *R. intraradices* inoculation; NM: without *R. intraradices* inoculation) and water treatment (WW: well-watered, 75% of the field water capacity; MD: mild drought, 50% of the field water capacity; ED: extreme drought, 25% of the field water capacity). We adopted a completely randomized block design with 6 treatments: NM + WW, AM + WW, NM + MD, AM + MD, NM + ED, and AM + ED. There were 3 replicates per treatment and 3 pots per replicate.

The sterilized cuttings were first grown in seedling pots under WW conditions for 3 weeks and then transferred to plastic pots containing 800 g of the culture substrate. At the same time, half of the plants were inoculated with *R. intraradices* (30 g/pot) while the other half were inoculated with equivalent amounts of sterilized inoculum. After 4 weeks of inoculation, 1/3 of both the inoculated and uninoculated plants was kept in the WW condition, 1/3 of both was treated with the MD condition, and the remaining 1/3 of both was treated with the ED condition. Soil moisture content was maintained by weighing at a fixed time (5:00 PM) every day. The experiment was carried out in a greenhouse (temperature: 25°C, relative humidity: 40%, light: 16 h/d, light intensity: 2,000 lx). The shoots and roots were separately harvested after 5 weeks of drought stress. Plant samples were carefully washed and rinsed 3 times with deionized water.

### Photosynthesis.

Three days before the experimental harvest, the leaf photosynthetic rate (Pn), stomatal conductance (Gs), intercellular CO_2_ concentration (Ci), and transpiration rate (Tr) were measured *in situ* on the third fully expanded leaf of each plant using a Li-6400 Portable Photosynthesis System (LI-COR Biosciences, Huntington Beach, CA). Measurements were performed in the morning (9:00 AM to 11:00 AM). We first checked the instrument before and after preheating, and then set the parameters as follows: photosynthetic photon flux density, 1,000 μmol·m^−2^ · s^−1^; constant airflow rate, 500 μmol · s^−1^; cuvette CO_2_ concentration, and 400 μmol CO_2_ · mol^−1^ air; we chose a standard 6-cm^2^ leaf chamber to measure the photosynthetic indices. Five measurements were performed on each leaf in three replicates after the CO_2_/H_2_O parameters became stable (37).

### Estimation of root colonization.

The root samples were rinsed with tap water, cut into approximately 1-cm sections, and then stained in 0.05% (wt/vol) trypan blue according to the methods of Phillips and Hayman (62). The stained root segments were spread on microscope slides and observed under a light microscope (Olympus BX43F, Tokyo, Japan). The AMF colonization rate was quantified using the magnified intersections method (63).

### Plant height, biomass, and root vitality.

Two days before experimental harvest, the height of each plant was measured and recorded. When harvesting, the above-ground part was cut, the root system was gently rinsed with tap water, and the washed root sample and above-ground part were weighed separately. A sample of about 1 g of fresh weight (FW) was used to measure the saturated fresh weight (SFW) after soaking in distilled water for 48 h, to determine the dry weight (DW) until it reached a constant weight in an oven at 70°C, and finally to measure the biomass of the above-ground and underground parts of the plant in proportion. The relative water content (RWC) of leaves was calculated according to Weatherley’s formula (64): RWC (%) = (FW – DW) × 100/(SFW – DW). Root activity was analyzed by the triphenyl tetrazolium chloride (TTC) method (65), expressed as TTC reduction intensity.

### Determination of antioxidant substances.

Frozen leaf or root tissue (0.1 g) was ground with 1 mL chilled buffer containing 50 mM potassium phosphate buffer (pH 7.8), 1 mM EDTA, 0.3% Triton X-100, and 1% (wt/vol) polyvinylpyrrolidone. The homogenate was centrifuged at 12,000 × *g* for 20 min at 4°C and the supernatant was used to conduct the enzyme assays. Superoxide dismutase (SOD) and peroxidase (POD) activity were measured according to the methods described by Beyer and Fridovich (66) and Amako et al. (67). The malondialdehyde content was measured using a UV-Vis spectrophotometer (UV-2550, Shimadzu Co. Ltd., Japan) using the method described by Kramer et al. (68) at wavelengths of 450, 532, and 600 nm.

### Determination of soluble protein, sugar, starch, and cellulose content.

The soluble protein in 0.05 g (fresh weight) of leaves or roots was determined by Coomassie bright blue G-250 staining. The soluble sugar, starch, and cellulose content of 0.02 g dry plant samples were determined by Gao’s method (69).

### Element concentration analysis.

The dried samples of leaves and roots were ground into a fine powder and passed through a 100-mesh sieve. Samples of 0.1 g of leaf or root were digested by microwave digestion (MA165-001 MultiPrep-41FC2) with 5 mL concentrated nitric acid, and then the volume was diluted to 50 mL with deionized water after removing the acid at 160°C. K, Ca, and Mg content was determined by flame atomic absorption spectrometry (70). P content was determined by the Mo-Sb colorimetric method (71).

### RNA extraction and RT-qPCR analysis.

The leaves and roots were thoroughly ground with liquid nitrogen and then extracted using the E.Z.N.A. Plant RNA kit (Omega Bio-Tek, GA, USA) to determine total RNA. The first-strand cDNA of 1 μg RNA was synthesized using a reverse transcription kit (HiScript II Reverse Transcriptase, Nanjing Vazyme Biotech Co., China). The 14-3-3 sequences of *P. cathayana* were cloned according to the *P. trichocarpa* genome database (Phytozome12) and the 14-3-3 gene sequence of *P. trichocarpa* (18). The cloning primers of *P. cathayana* 14-3-3 family gene sequences are shown in Table S1 in the supplemental material. Phylogenetic analysis and multiple sequence comparison analysis were performed to determine the 14-3-3 sequence of *P. cathayana*. At the same time, the 14-3-3 sequence of *R. intraradices* was downloaded from the NCBI database.

The reverse transcription cDNA was diluted 5 times for qRT-PCR analysis. The primers for qRT-PCR are shown in Table S2 in the supplemental material. The reaction system consisted of 10 μL: 5 μL 2× ChamQ SYBR qPCR Master Mix (Vazyme), 1 μL upstream primer, 1 μL downstream primer, and 2 μL sterile water. The reaction conditions were as follows: 95°C for 3 min; 39 cycles at 95°C for 10 s, 58°C for 20 s, and 72°C for 20 s; followed by 95°C for 10s, and then 61 cycles of 5 s each increasing from 65°C to 95°C in increments of 0.5°C for each cycle, and the specific annealing temperature for each gene needed to be changed. *PcGLL* and *Pctublin* were the internal reference genes of *P. cathayana* qRT-PCR and *RiEF1α* and *Ritublin* were the internal reference genes of *R. intraradices*. The cycle threshold (2^–ΔΔCT^) method (72) was used to calculate the relative quantitative values of different genes, and a dissociation curve analysis was used to determine the specificity of primer amplification.

### Statistical analysis.

IBM SPSS Statistics software version 26.0 (SPSS Inc., Chicago, IL, USA) was used to analyze the experimental data. An independent-sample Duncan test and a two-way analysis of variance (ANOVA; factors: AMF inoculation and drought stress) (*P* < 0.05) were used to compare differences among treatments. All data were reported as means ± standard deviation (SD). MAGA6 was used to construct a phylogenetic tree using the neighbor-joining method (bootstrap = 1,000). The orthogonal partial least-squares discrimination analysis data for different metabolites and gene expression were analyzed using the Wekemo Bioinformatics cloud (https://www.bioincloud.tech). Correlation heatmap analysis, redundancy analysis (RDA), and principal-component analysis (PCA) were also performed using the Wekemo Bioinformatics cloud.
