# Supplementary material for: Arbuscular Mycorrhizal Fungi Enhanced Drought Resistance of Populus cathayana by Regulating the 14-3-3 Family Protein Genes
Source: Microbiol Spectr. 2022 May 25;10(3):e02456-21. doi: 10.1128/spectrum.02456-21 (PMC9241863; doi:10.1128/spectrum.02456-21)
Supplement: SUPPLEMENTAL FILE 1 — Fig. S1 to S4 and Tables S1 and S2. Download spectrum.02456-21-s001.pdf, PDF file, 0.6 MB [file spectrum.02456-21-s001.pdf]

Table S1 Cloning primer of 14-3-3 protein gene from *Populus cathayana* and *R.intraradices*

Gene	Forward Primers (5'-3')	Reverse Primers (5'-3')
PcGRF1	ATGGCTGTCACACCATC	TCACTGCTGTTCATCACC
PcGRF2	ATGGCAGCCACACCATCTGCTC	TCACTGTTCATCACCAGTT
PcGRF3	ATGTCGCCAACTGAATCATC	TTACACAAATGTGCAAATGG
PcGRF4	ATGTCTCCAACTGAACCATC	TCACTGCGGCCCATCGCCTGATT
PcGRF5	ATGTCTCCAACTGAACCATC	AGAGAGAGAGAGAGAGAGAG
PcGRF6	ATGGCAACCACCACCACTG	CTATGGCTCATCTAACTGG
PcGRF7	ATGGTTACCACCACCACTG	CTATAAGCTAACACAGAGAG
PcGRF8	ATGGATTCTTCAAAAGATCGCG	TCACTCGGCATCTTCACCCTC
PcGRF9	ATGGATTCATCAAAGGATCG	TCACTCAGCATCTTCACCC
PcGRF10	ATGGAGAAGGAGAGAGAGCAG	CTATTTCTCTGCCCTGGGTTC
PcGRF11	AATCTGGTGTCCGTGGGT	TGAGGCTATCAAGTTCTGCT
PcGRF12	ATGTCAACCGAGAAGGAG	TCAGTGCTCTCCTTCGGCAGG
PcGRF13	ATGTCGACCGATAAGGAGAGAG	TCAGTGCTCTCCTTCGGCAGG
PcGRF14	GAAACGCTACAATGAGAT	AGTATTGGGATAGCCTCA
Ri14-3-3	GGAACGCGAGAACCAGAC	GAAGTCCAAAGGGTCAAG

Table S2 Gene-specific primers used for qRT-PCR.

Gene	Forward Primers (5'-3')	Reverse Primers (5'-3')
PcGLL	CTCTCATTGAGCCGGCAAAT	CCCCCCTTCAAGCATAAGG
Pctublin	GATTTGTCCCTCGCGCTGT	TCGGTATAATGACCCTTGGCC
PcGRF1	ATTGGCTCTTAACTTCTCTGTCTTC	ACGTATCCAGCTCCGCAATG
PcGRF2	CCAACGGCTTCTTCTGGTGA	GAGACGGATTGGGTGAGTGG
PcGRF3	TTGCCAAGCAGGCTTTTGAC	AGTGTGCAACCTTGGGGAAA
PcGRF4	TCTCCAACTGAACCCATCACG	ATCCTCCATGAAGCCCTCCT
PcGRF5	GAAGCTGGGCTCAGCAAGAT	TGCTCTCAGCAGCTTCCTTC
PcGRF6	TCTGGCTGAGTTCAAGGCTG	CCTCAAATGCCTGTTTCGCC
PcGRF7	CTGATGTTTGTGCTAGTATTTTGAGGT	GCAATATCCTGAGCAGCCTTG
PcGRF8	ATACCCGGGGAATCCTCTGT	GCCCGTTCAGGAGAGTTCAT
PcGRF9	TTCGACTGGGTTTGGCTCTT	TCTGGTCTTCTCCATCCTCAG

PcGRF10	CCTGGCACTCAACTTTTCCG	CTGTAGAGTGCTCACCTCCTTC
PcGRF11	AACTCCCCTGAAAGGGCCTG	TCAGTTTTGCTTTACCTCTGCC
PcGRF12	CCGAGCGCTATGACGAGA	AGACATGATCCGCCAAGAAGC
PcGRF13	GCGCTATGATGAAATGGTTGAGAG	CTGGCAGTAACCCTTGATCAGT
PcGRF14	CTCCCTTCAACTTCCCAGTGT	GCCAACTTGGATGCAGCTTT
RiEF1α	TGTTGCTTTCGTCCCAATATC	GGTTTATCGGTAGGTCGAG
Ritublin	TGTCCAACCGGTTTTAAAGT	AAAGCACGTTTGGCGTACAT
Ri14-3-3	GGTAATGAAACCCAAGTTCAG	GCGATATCTGTTGCGTGTTTA

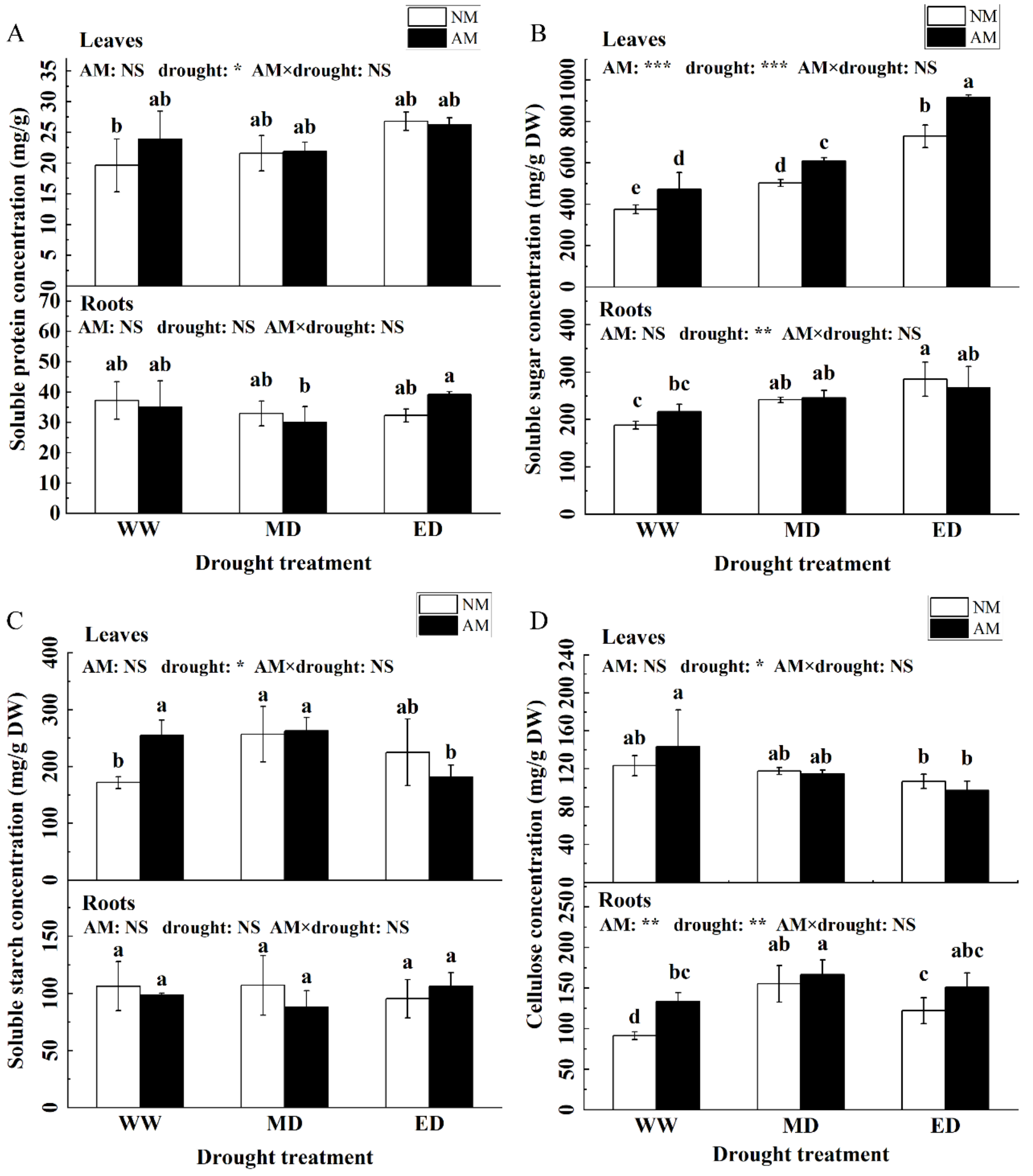

Fig. S1: Effect of AMF inoculation on the soluble substance of *P. cathayana* under drought stress (mean ± SD, n =

3).

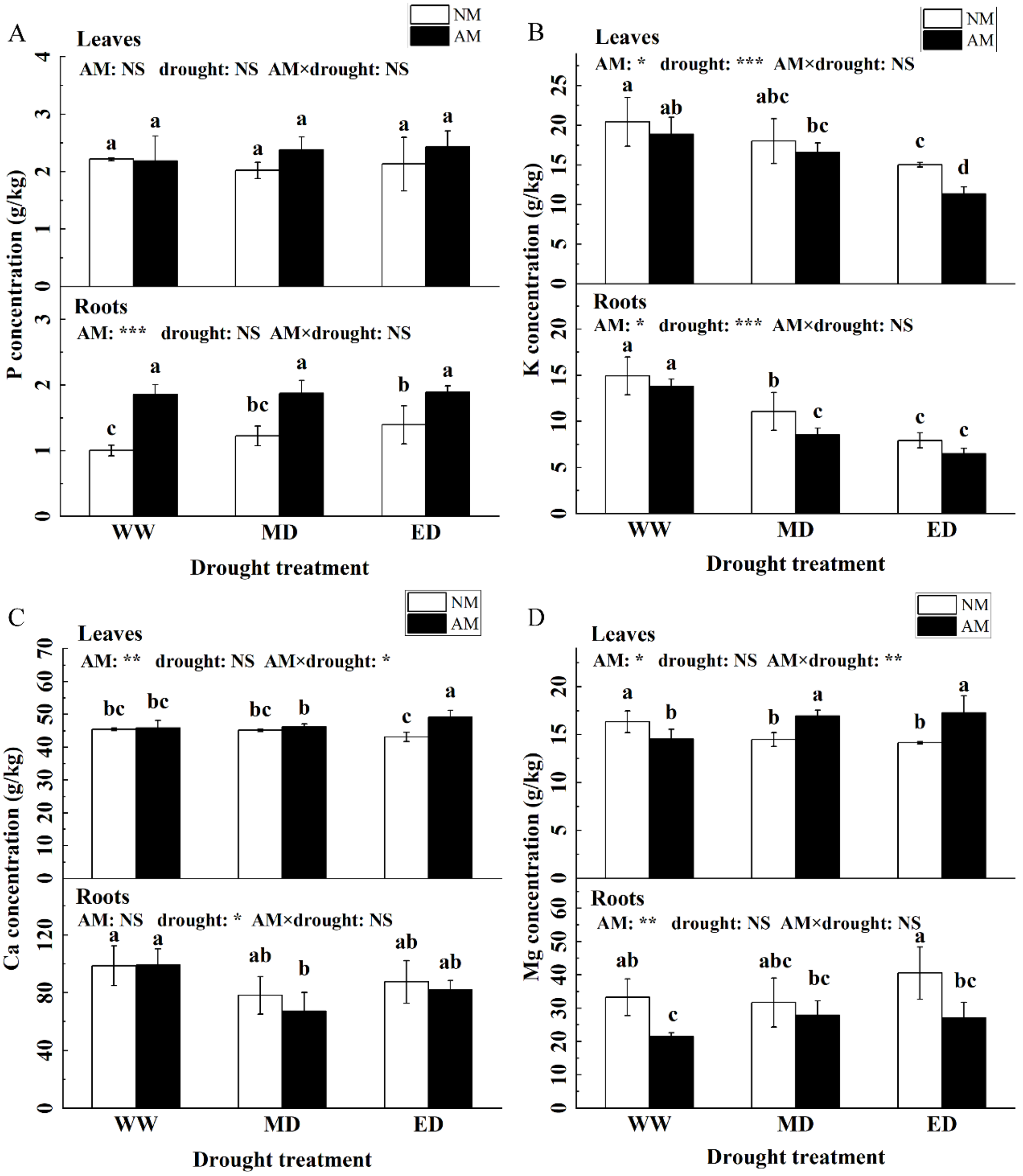

Fig. S2: Effect of AMF inoculation on the nutrient element content of *P. cathayana* under drought stress (mean ± SD, n = 3).

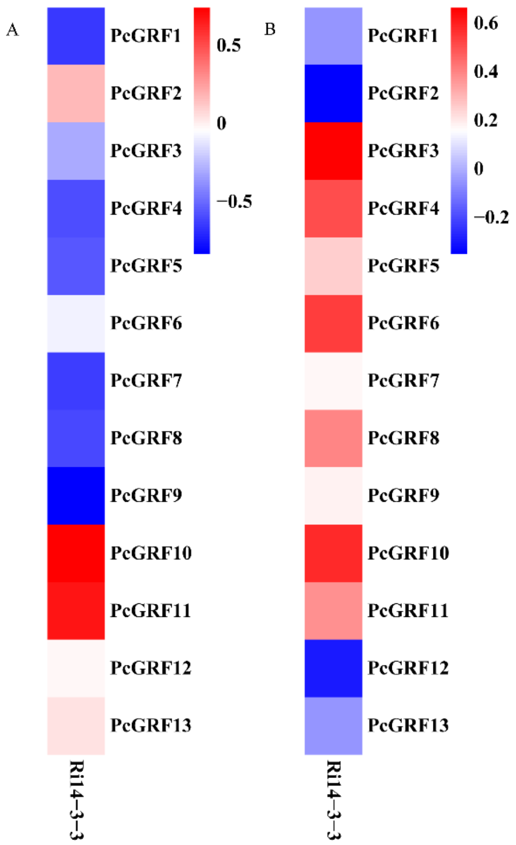

Fig. S3: Correlation heatmap (A: leaf; B: root) of *Ri14-3-3* of *R. intraradices* and 14-3-3 gene expression of *P. cathayana*.

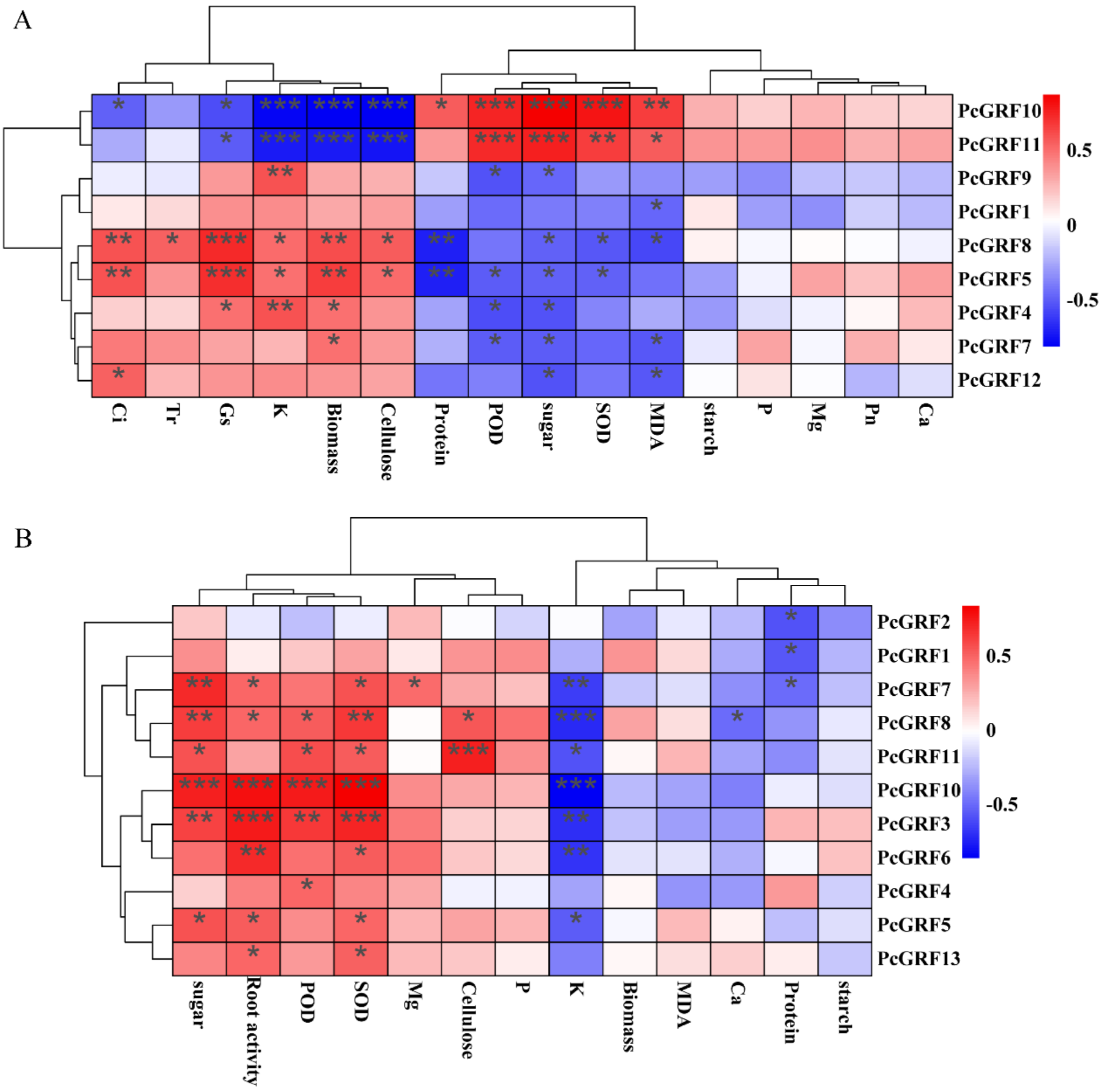

Fig. S4: Correlation heatmap (A: leaf; B: root) of gene expression and physiological indicators of *P. cathayana* under different drought and inoculation conditions. *P <0.05, **P <0.01 and ***P <0.001.
